# 

**Published:** 1914-10-17

**Authors:** 


### CONTENTS.

PAGE

**The Role of the Physician in Time of War** ... 55

**The Tuberculosis Danger Among Recruits** ... 56

**Hospital and Institutional News** ... ... ... 57

Cumberland Infirmary, Carlisle—The Moral of the Tragedy at Barnet Infirmary—Advanced Consumptives and Kitchener's Army—Consumptive Recruits in Military Hospitals—Improvements at the North Evington Infirmary—A Hospital's Local Reputation — Leeds Memorial to Dr. E. F. Trevelyan—A Thrilling Story of the Loss of the *Aboukir*—A Hospital Chairman for the Front—The Hospital Barge—Mental Hospital Staffs and the War—The Salary of a Deputising Medical Officer—Radium for Poor-Law Infirmaries—The Ceremony of Circumcision on the Day of Atonement—Leicester's Poor Record for Hospital Sunday—The New Birmingham Sanatorium—Wandsworth and the War—Postponement of the Revision of Medical Benefit Regulations—A Change in the Rules at Territorial Hospitals—A Hospital for the Indian Troops—Classification of Workhouse Inmates in Shropshire—The Weak Point in the Scheme—Panel Practitioners and Free Choice of Chemist—Institutional Pharmacists' Winter Programme—Alexandra Day in Leicester—Chocolate and Tobacco as Church Decorations—A September Record—A Manchester Hospital and the War,

PAGE

**With a British Field Ambulance in August** ... 63

By an Army Medical Officer at the Front.

**Buildings Contemplated** ... ... ... ... ... 65

**Round the World** ... ... ... ... ... 66

Fellow-Workers and the Roll of Honour.

**The Making of a Territorial Hospital** ... ... 76

The Value of Active Co-operation by Civilians.

**Medical Appointments** ... ... ... ... ... 68

**Hospitals and the War** ... ... ... ... ... 69

**Latest Arrivals in London Hospitals**

The Wounded at St. George's Hospital—Croydon

**The Care of the Soldiers in Provincial Hospitals**

Cambridge: The New War Hospital—Cardiff: Where the Men like to Convalesce—

[See over.]

### CONTENTS—(*continued*).

PAGE

Coventry: The First Batch of Wounded—Gravesend: Belgian Wounded Arrive—Leicester: The County Regiment—Sheffield: The Amusements of Convalescence—Torbay Hospital and Wounded Belgians—Tunbridge Wells: New Arrivals and New Funds—Wakefield: The Refugee Question—Weston: The Special Care of Convalescent Soldiers.

**The Editor's Letter-Box** ... ... ... ... 72
The Use of the Red Cross.
The Needs of Wounded Women.
Proposed Special Service (Cholera) Corps.

PAGE

**Radio-Therapeutic Department at New King's** 73
Official Account of its Plan and Equipment.

**Experiences on H.M.S. "Aboukir"** ... ... 76

The Action of Torpedoes and Submarines.

**The London Panel Committee: Tuesday's Decisions** ... ... ... ... ... ... 76

**The Institutional Worker** ... ... (Suppl.) 1
War Matters.
Fire Brigades and Hospitals.
Questions for October.
Questions Invited.
The Sick and in Need.
Employment and Training.
Miscellaneous.

